# *De novo* comparative transcriptome analysis provides new insights into sucrose induced somatic embryogenesis in camphor tree (*Cinnamomum camphor*a L.)

**DOI:** 10.1186/s12864-015-2357-8

**Published:** 2016-01-05

**Authors:** Xueping Shi, Cuijie Zhang, Qinhong Liu, Zhe Zhang, Bo Zheng, Manzhu Bao

**Affiliations:** Key Laboratory of Horticultural Plant Biology of Ministry of Education, College of Horticulture and Forestry Sciences, Huazhong Agricultural University, Wuhan, 430070 P. R. China

**Keywords:** *Cinnamomum camphora* L, Somatic embryogenesis, RNA-seq, *De novo* assembly, Comparative transcriptome, Osmotic stress

## Abstract

**Background:**

Somatic embryogenesis is a notable illustration of cell totipotency, by which somatic cells undergo dedifferentiation and then differentiate into somatic embryos. Our previous work demonstrated that pretreatment of immature zygotic embryos with 0.5 M sucrose solution for 72 h efficiently induced somatic embryo initiation in camphor tree. To better understand the molecular basis of somatic embryogenesis induced by osmotic stress, *de novo* transcriptome sequencing of three tissues of camphor tree (immature zygotic embryos, sucrose-pretreated immature zygotic embryos, and somatic embryos induced from sucrose-pretreated zygotic embryos) were conducted using Illumina Hiseq 2000 platform.

**Results:**

A total of 30.70 G high quality clean reads were obtained from cDNA libraries of the three samples. The overall *de novo* assembly of cDNA sequence data generated 205592 transcripts, with an average length of 998 bp. 114229 unigenes (55.56 % of all transcripts) with an average length of 680 bp were annotated with gene descriptions, gene ontology terms or metabolic pathways based on Blastx search against Nr, Nt, Swissprot, GO, COG/KOG, and KEGG databases. CEGMA software identified 237 out of 248 ultra-conserved core proteins as ‘complete’ in the transcriptome assembly, showing a completeness of 95.6 %. A total of 897 genes previously annotated to be potentially involved in somatic embryogenesis were identified. Comparative transcriptome analysis showed that a total of 3335 genes were differentially expressed in the three samples. The differentially expressed genes were divided into six groups based on K-means clustering. Expression level analysis of 52 somatic embryogenesis-related genes indicated a high correlation between RNA-seq and qRT-PCR data. Gene enrichment analysis showed significantly differential expression of genes responding to stress and stimulus.

**Conclusions:**

The present work reported a *de novo* transcriptome assembly and global analysis focused on gene expression changes during initiation and formation of somatic embryos in camphor tree. Differential expression of somatic embryogenesis-related genes indicates that sucrose induced somatic embryogenesis may share or partly share the mechanisms of somatic embryogenesis induced by plant hormones. This study provides comprehensive transcript information and gene expression data for camphor tree. It could also serve as an important platform resource for further functional studies in plant embryogenesis.

**Electronic supplementary material:**

The online version of this article (doi:10.1186/s12864-015-2357-8) contains supplementary material, which is available to authorized users.

## Background

Camphor tree (*Cinnamomum camphora* L), a broad-leaved evergreen tree within the Lauraceae family, native to China and Japan, is now cultivated in many countries as an ornamental plant or as a source of camphor [1]. The species is also considered as a medicinal plant in the treatment of muscular strains, inflammation, and rheumatic conditions and has antiseptic properties. Plant regeneration system for camphor tree has been established via direct somatic embryogenesis [2, 3], in which immature zygotic embryos (IZE) were collected and pretreated with 0.5 M sucrose solution before somatic embryo (SE) induction. Sucrose pretreatment, other than various plant hormones, significantly enhanced efficiency of SE initiation from 16.29 to 93.27 % in this recalcitrant species [3].

Somatic embryogenesis is a notable illustration of cell totipotency, by which somatic cells undergo dedifferentiation and then differentiate into somatic embryos [4]. The developmental pathway of somatic embryos shares high similarities at almost all developmental stages to that of their zygotic counterparts, which makes it an attractive model system to study zygotic embryogenesis at molecular, cellular, and tissue levels [5, 6]. Somatic embryogenesis has also been considered as a potential model system for studying developmental mechanism of early embryogenesis [7, 8].

The initiation of SE is a multi-factorial event, in which embryos are derived from vegetative cells by exposing explants to stress conditions or exogenous growth regulators. It is widely recognized that plant hormones, particularly auxin, are the most important factor in stimulating SE initiation, and stress is another factor becoming increasingly recognized in recent years [8]. The stress factors which can stimulate initiation of embryogenic competence include heavy metal ions [9, 10], high temperature [11, 12], explant wounding [13], and high osmotic stress [3, 14–16].

Though initiation of somatic embryogenesis has been observed in many species, the molecular mechanism of triggering vegetative-to-embryogenic transition remains a challenge [17]. It is believed that somatic embryogenesis is a developmental process involving gene expression reprogramming that engages a cascade of genetic triggers turning on or off the expression of specific genes [18, 19]. Analyses of gene expression during somatic embryogenesis can provide information for better understanding of this complicated process.

The patterns of gene regulation during somatic embryogenesis have been investigated in several species, including carrot [20], Arabidopsis [21], alfalfa [22], soybean [23–25], cotton [26, 27], potato [28] and orange [29]. Numerous specifically activated or differentially expressed genes related to somatic embryogenesis have been isolated, such as *Somatic Embryogenesis Receptor-like Kinase* (*SERK*), *Leafy Cotyledon* (*LEC*), *Baby Boom* (*BBM*), and *Wuschel* (*WUS*) (reviewed by [30]). Genes controlling early embryogenesis, including *Auxin Response Factor19* (*ARF19*), *WUS*, *LEC1*, *SERK1* and *Heat Shock Protein 17* (*HSP17*) have been investigated (reviewed by [31]). Molecular basis of stress-induced acquisition of embryogenic competence was described in detail by Karami and Saidi [8]. However, most of these researches have been focused on gene regulation in the process of somatic embryo initiation induced by plant hormones. The role of stress, especially high osmotic stress, in embryogenic culture has not been well characterized at molecular level in plants, including camphor tree.

Camphor tree is a species lacking genome resources and a comprehensive investigation of the global transcription. In an attempt to understand the molecular basis of embryogenic competence acquisition and SE formation in camphor tree, we separately performed *de novo* transcriptome sequencing of IZE, IZE pretreated with 0.5 M sucrose solution for 72 h (IZE_Suc), and SE obtained from cultured IZE_Suc (SE_5w) using Illumina Hiseq 2000 technology. This work provides new insights into somatic embryogenesis induced by high osmotic stress, and valuable resources for future transcriptomic, genomic and genetic research on camphor tree. The results in the present study also lead to the hypothesis that sucrose induced somatic embryogenesis may share or partly share the mechanisms of somatic embryogenesis induced by plant hormones.

## Methods

### Plant materials

Immature fruits were collected in late July of 2013 (12–13 weeks after open pollination) from a mature camphor tree in the campus of Huazhong Agricultural University, Wuhan, China. The fruits were surface-sterilized with 0.1 % (w/v) mercuric chloride (HgCl_2_) solution for 10 min, and then rinsed three times with sterilized distilled water. The fruits were cut open, and IZEs were isolated carefully from the distal end of the fruits (Additional file 1: Figure S1A-C). Meanwhile, IZEs were pretreated with 0.5 M sucrose solution for 72 h. SE_5w were obtained by culturing the sucrose-pretreated IZEs on hormone-free induction medium for five weeks, which contained cotyledonary SE as well as global, heart-shaped and torpedo SE since the asynchronous secondary somatic embryogenesis (Additional file 1: Figure S1D-E) [2]. IZE isolation, sucrose pretreatment, and somatic embryo induction from pretreated IZEs were carried out according to the method previously described [3]. According to our previous results, pretreatment of IZEs with 0.5 M sucrose solution could significantly improve the frequency of SE initiation [3]. In this study, three samples, namely IZE, sucrose-pretreated IZEs (IZE_Suc), and SE_5w, were collected and frozen immediately in liquid nitrogen and then kept at −80 °C for transcriptome analysis. Both samples of IZE and IZE_Suc in the weight of 300 mg were mixed sample pools composed of approximately 2000 embryos about 2 mm in diameter. The sample of SE_5w (300 mg) was also a mixed sample pool containing SEs in different developmental stages, which consisted of about 200 SEs. In addition, stems, fully expanded young leaves, young flowers, and young fruits were collected during March and May. Mature seeds were separated from mature fruits which were collected in November. Roots were obtained from germinated seeds. These samples were also collected and frozen immediately in liquid nitrogen and kept at −80 °C for the experiments of quantitative Real-Time PCR (qRT-PCR). All samples were collected from a single tree in the same year.

### RNA isolation

Each frozen sample was ground in a mortar with liquid nitrogen. Total RNA was isolated using Trizol reagent (Invitrogen, Shanghai, China) according to the manufacturer’s instruction. RNA purity was checked using the NanoPhotometer® spectrophotometer (Implen, CA, USA), RNA concentration was measured using Qubit^®^ RNA Assay Kit in Qubit^®^ 2.0 Flurometer (Life Technologies, CA, USA), and RNA integrity was assessed using the RNA 6000 Nano Kit of the Bioanalyzer 2100 system (Agilent Technologies, CA, USA).

### Transcriptome sequencing and *de novo* assembly

A total amount of 3 μg RNA with RIN value above 8.0 from each of the three samples was respectively used to generate sequencing library using Illumina TruSeq™ RNA Sample Preparation Kit (Illumina, CA, USA) following manufacturer’s recommendations. Six index codes were added to each sample for attributing sequences. The clustering of the coded samples was performed on a cBot Cluster Generation System using TruSeq PE Cluster Kit v3-cBot-HS (Illumina) according to the manufacturer’s instructions. After cluster generation, the libraries were sequenced on an Illumina Hiseq 2000 platform to generate 100 bp paired-end reads.

The clean reads were obtained from raw data by filtering out adapter-only reads, reads containing poly-N, and low quality reads. The values of Q20, Q30, GC-content and sequence duplication level of the clean data were calculated. Clean reads were then assembled with the Trinity program [32]. In order to ensure the quality of assembly, the reads were mapped back to the assembled transcripts using the bowtie aligner by Visualization and Quality Assessment application within Trinity software. The alignment was visualized with Integrated Genomics Viewer (IGV) version 2.3.2 [33]. CEGMA software [34] was used to assess the sequence completeness of the assembly by estimating the presence and completeness of 248 ultra-conserved eukaryotic genes. Profile-hidden Markov model was used to ensure reliability of gene structure.

### Gene functional annotation and classification

Unigene sequences were aligned using Blastx with an E-value cut-off of 1.0e-5 (unless stated otherwise) against protein databases, with the priority order of NCBI Nr (non-redundant database), Nt, Swiss-Prot, KEGG (Kyoto Encyclopedia of Genes and Genomes, E-value = 1.0e-10), and KOG/COG (clusters of orthologous groups, E-value = 1.0e-3) if conflicting results were obtained, to retrieve proteins with the highest sequence similarity with the given unigenes along with their functional annotation. Based on the Nr annotation, GO (gene ontology) annotation (E-value = 1.0e-6) was generated using Blast2GO program [35], and GO functional classification was finished using the WEGO software [36].

### Analysis of differential gene expression

Gene expression levels were estimated by mapping clean reads to reference set of assembled transcripts using RSEM [37] for each sample. RPKM (reads per kilo bases per million mapped reads) were used as the value of normalized gene expression levels [38]. Pairwise differential expression analysis was done among the three samples using DEGseq [39] R package. P-values were adjusted using the q value method proposed by Storey et al. [40]. A q value < 0.005 and an absolute value of log2 fold_change >1 provided thresholds to determine significant differences in gene expression. Go enrichment analysis (p-value ≤ 0.05) of the differentially expressed genes (DEGs) was performed using GOseq with the Wallenius non-central hyper-geometric distribution model [41] to adjust gene length bias in DEGs. KEGG pathway enrichment analysis of the DEGs was done using KOBAS [42] with the hyper-geometric distribution model. The enrichment p-values were adjusted using the Benjamin and Hochberg method.

### Quantitative Real-Time PCR (qRT-PCR) analysis

Analysis of qRT-PCR was performed to validate gene expression results from RNA-seq. Total RNA (3 μg) from each sample was reverse-transcribed into single-stranded cDNA using Prime-Script^TM^ RT reagent Kit (TaKaRa, Dalian, China). Mixture of PCR was composed of 10 μl 2× SYBR Premix DimerEraser (TaKaRa, Dalian, China), 1 μl of each primer (Additional file 2: Table S1), and 2 μl of cDNA diluted 1:50. PCR reactions were run on an ABI 7500 Real-Time System (PE Applied Biosystems, CA, USA) under the following conditions: initial incubation at 50 °C for 2 min and 95 °C for 30 s, followed by 40 cycles of 95 °C for 15 s and 60 °C for 1 min. Gene expression and standard error were calculated over three biological and two technical replicates.

## Results

### *De novo* assembly of *camphor tree* transcriptome

**Fig. 1:**
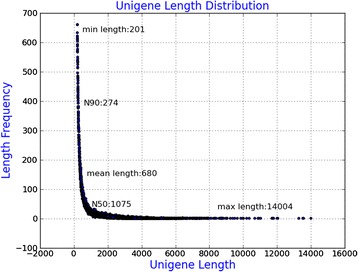
Length distribution of unigenes in the assembled transcriptomes. The x axis shows the lengths of unigenes and the y axis shows the number of unigenes

Genome and transcriptome resources are scarce for gene functional analysis in camphor tree. In this study, *de novo* transcriptome assembly was performed by merging the valid reads from libraries of three types of camphor embryos, including IZE, IZE_Suc and SE_5w. Sequencing of cDNA libraries from the three samples resulted in 104,771,602, 84,950,386 and 117,117,800 clean reads, representing with 10.48G, 8.50G and 11.72G nucleotides, respectively (Table 1). The statistics of raw reads were also shown in Table 1. For all the sequence data, Q20 percentage was more than 90 %, while Q30 percentage was more than 80 % (Table 1). A total of 306,839,788 clean reads obtained from 319,668,420 raw reads (95.99 %) participated in the assembly (Table 1). The overall *de novo* assembly of cDNA sequence data generated 205592 transcripts, with an average length of 998 bp. The reads were assembled into 114229 non-redundant unigenes with an average length of 680 bp and an N50 of 1075 bp. All the unigenes were longer than 200 bp in length, 71566 of them (62.65 %) were 200 to 500 bp, and 7375 (6.46 %) were longer than 2 kb (Additional file 3: Table S2, Fig. 1).

**Table 1:** Overview of output statistics on camphor tree transcriptome sequencing

Parameter	IZE	IZE_Suc	SE_5w
The number of total raw reads	108,397,716	89,960,198	121,310,506
The number of total clean reads	104,771,602	84,950,386	117,117,800
Total base pairs (bp) of clean reads	10.48G	8.5G	11.72G
Q20 percentage	96.19 %	93.21 %	96.25 %
Q30 percentage	89.03 %	82.76 %	89.50 %
N percentage	0.05 %	0.05 %	0.05 %
GC percentage	46.85 %	45.40 %	46.12 %

Q20/30 percentage represents proportion of nucleotides with quality value larger than 20/30, and N percentage represents proportion of unknown nucleotides in clean reads

To assess the quality of the assembled transcripts, the clean reads were mapped back to the assembly, and the results showed that 82.73 % of the clean reads could be mapped back on the assembled transcriptome, among which 47.33 % were mapped uniquely and 35.4 % were mapped more than once. Assessment of assembly completeness by CEGMA software showed 237 out of 248 ultra-conserved core proteins were ‘complete’ in the transcriptome, yielding a completeness of 95.6 %. Five genes were identified as ‘partial’ genes. These results indicated high quality of transcriptome assembly in this study.

### Functional annotation of non-redundant unigenes

To annotate the Trinity-assembled unigenes, the 114229 unigenes were subjected to Blastx searches against seven public databases, returning an above cut-off BLAST result (Table 2). A total of 26640 (23.32 %), 10741 (9.4 %), 19352 (16.94 %), 27673 (24.22 %), 32920 (28.81 %) and 11281 (9.87 %) of the unigenes were annotated by Nr, Nt, Swiss-Prot, PFAM, GO and KOG/COG database, respectively (Additional file 4: Table S3). 7742 (6.77 %) of the unigenes were annotated by KEGG database (Additional file 5: Table S4). Among the unigenes, 38020 unigenes (33.28 % of all unigenes) were annotated by at least one database. Of them, 3282 (2.87 %) of the unigenes were simultaneously annotated by all databases.

**Table 2:** Annotation of assembled camphor tree unigenes

Database for annotation	Number of annotated unigenes	Percentage (%)
Nr	27752	23.33
Nt	10741	9.4
SwissProt	19352	16.94
PFAM	27673	24.22
KOG	11281	9.87
GO	32920	28.81
KEGG	7742	6.77
Annotated in all Databases	3282	2.87
Annotated in at least one Database	38020	33.28
Total queries/unigenes	114229	100

**Fig. 2:**
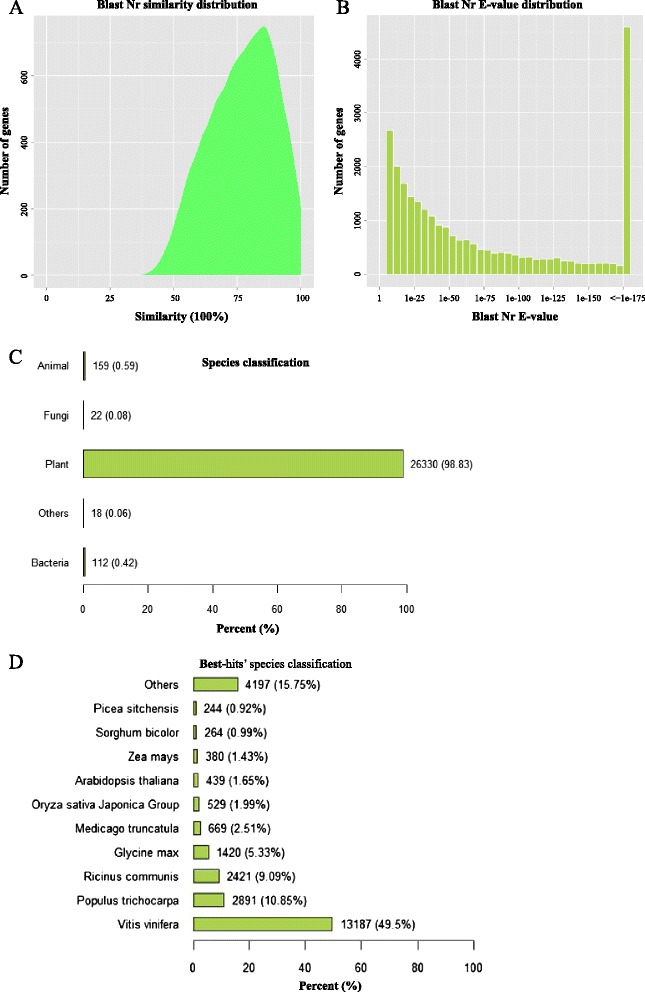
Further analyse of the BLAST results in Nr database. **a** Similarity distribution; **b** E-value distribution; **c** Best hit species distribution; **d** Best hit species classification

To further analyze the BLAST results in Nr database, similarities distribution, E-value distribution, best-hit species distribution, and best-hit species classification were investigated. The results showed that 3330 (12.50 %) of the matched sequences had alignment identities greater than 85 % (Fig. 2a). Only 2661 (9.99 %) of the matched sequences had an E-value higher than 1.0e-10, and 14848 (55.73 %) of them had an E-value lower than 1.0e-50 (Fig. 2b), which indicated high-reliability of the alignment. Among the annotated unigenes, the majority (26330, 98.83 %) matched plants (Fig. 2c). The top three matched plant species were *Vitis vinifera* (13187, 49.5 %), *Populus trichocarpa* (2891, 10.85 %), and *Ricinus communis* (2421, 9.09 %) (Fig. 2d).

With respect to somatic embryo initial function, 897 unigenes were identified as homologues to the previously annotated genes that are potentially involved in somatic embryogenesis (Additional file 6: Table S5). Among them, *Heat-Shock proteins* (*HSPs*, 296 unigenes) were the most dominant group, followed by *Chitinase* genes (73 unigenes), *Ethylene Responsive Factor* (*ERF*, 61 unigenes), *Glutathione S-Transferase* (*GST*, 59 unigenes), *Auxin Responsive Factor* (*ARF*, 44 unigenes), and *C-Repeat Binding Factor*/*Dehydration-Responsive Element-Binding Protein* (*CBF*/*DREB*, 41 unigenes) (Additional file 6: Table S5).

### Classification of camphor tree unigenes

**Fig. 3:**
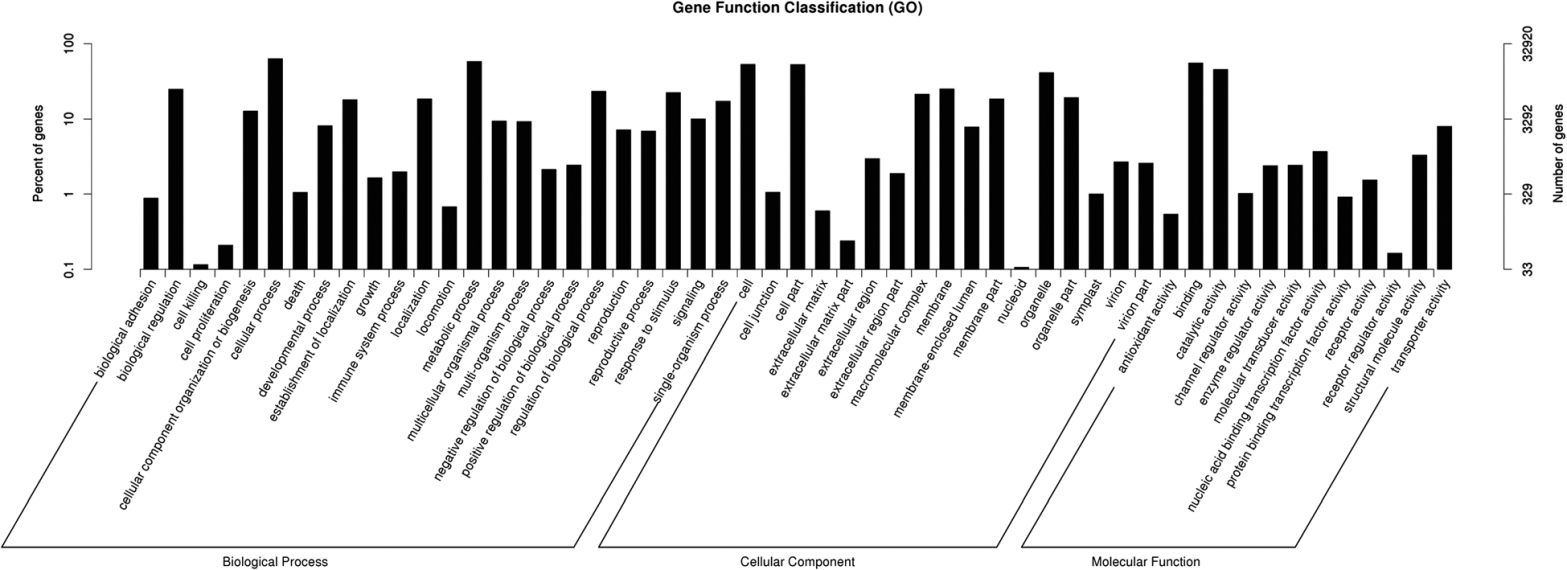
GO classifications of assembled unigenes by Blast2GO in Camphor tree. Unigenes were annotated in three main categories: biological process, cellular component and molecular function. The x-axis indicates the sub-categories and the y-axis indicates the number of unigenes

GO assignment was performed to classify the functions of predicted unigenes. The 32920 unigenes annotated in GO database were categorized into 57 functional groups, belonging to three main GO ontologies: biological process, cellular component, and molecular function (Fig. 3). Among the functional groups, “cellular process”, “metabolic process”, “binding”, “cell”, and “cell part” terms were dominant (Fig. 3).

**Fig. 4:**
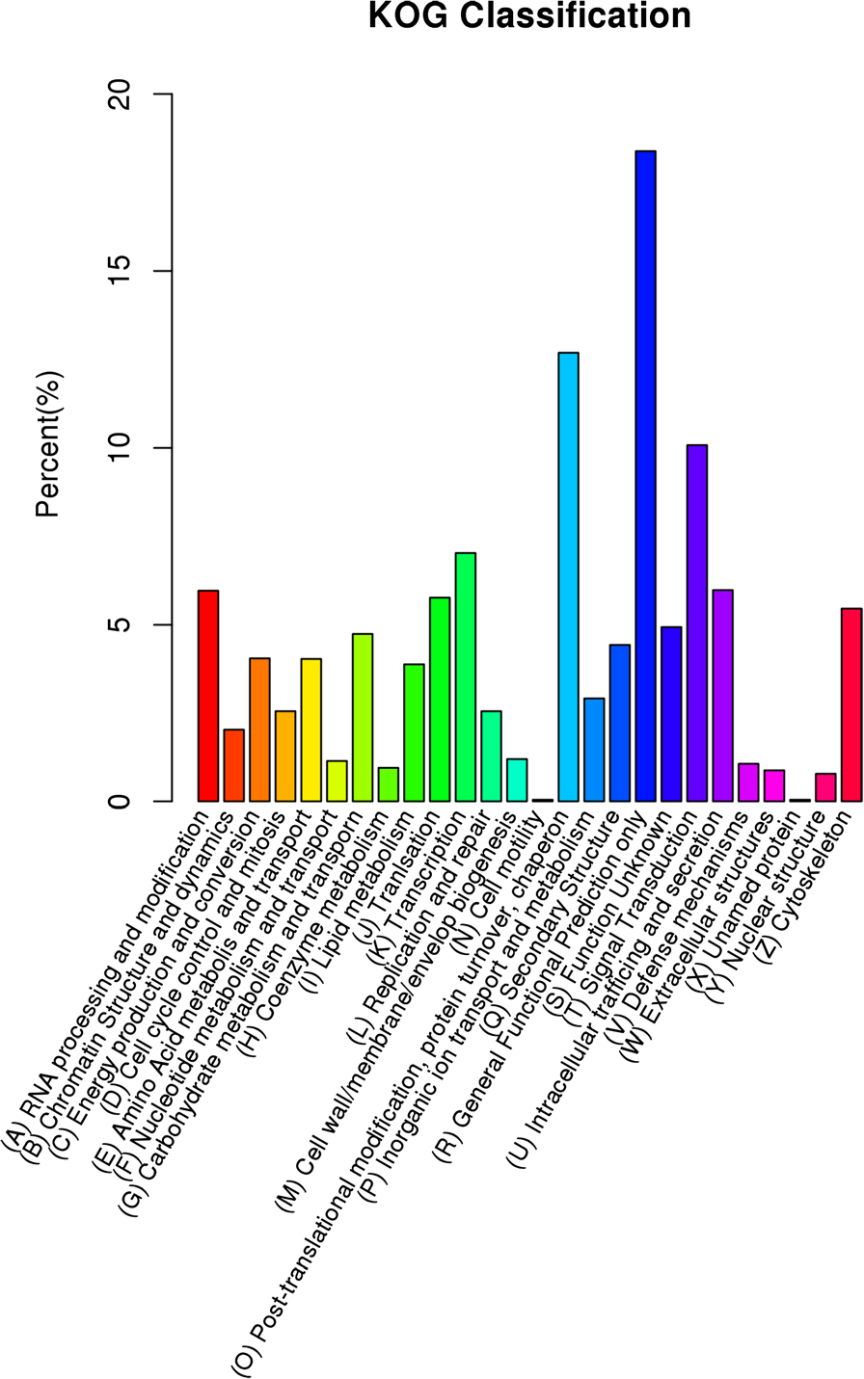
KOG classifications of assembled unigenes in Camphor tree. Out of 114229 *de novo* assembled unigenes, 11281 were annotated and seperated into 26 categories

To further evaluate the function of the assembled unigenes, we searched the annotated unigenes involved in COG. COG annotation yielded 11281 putative proteins in 26 categories (Fig. 4). Among these categories, the cluster for “general functional prediction only” (18.39 %) represented the largest group, followed by “post-translational modification, protein turnover, chaperon” (12.69 %), “signal transduction” (10.08 %) and “transcription” (7.02 %). Clusters for “cell motility” (0.04 %), “unnamed protein” (0.04 %) and “nuclear structure” (0.78 %) were the smallest groups (Fig. 4).

**Fig. 5:**
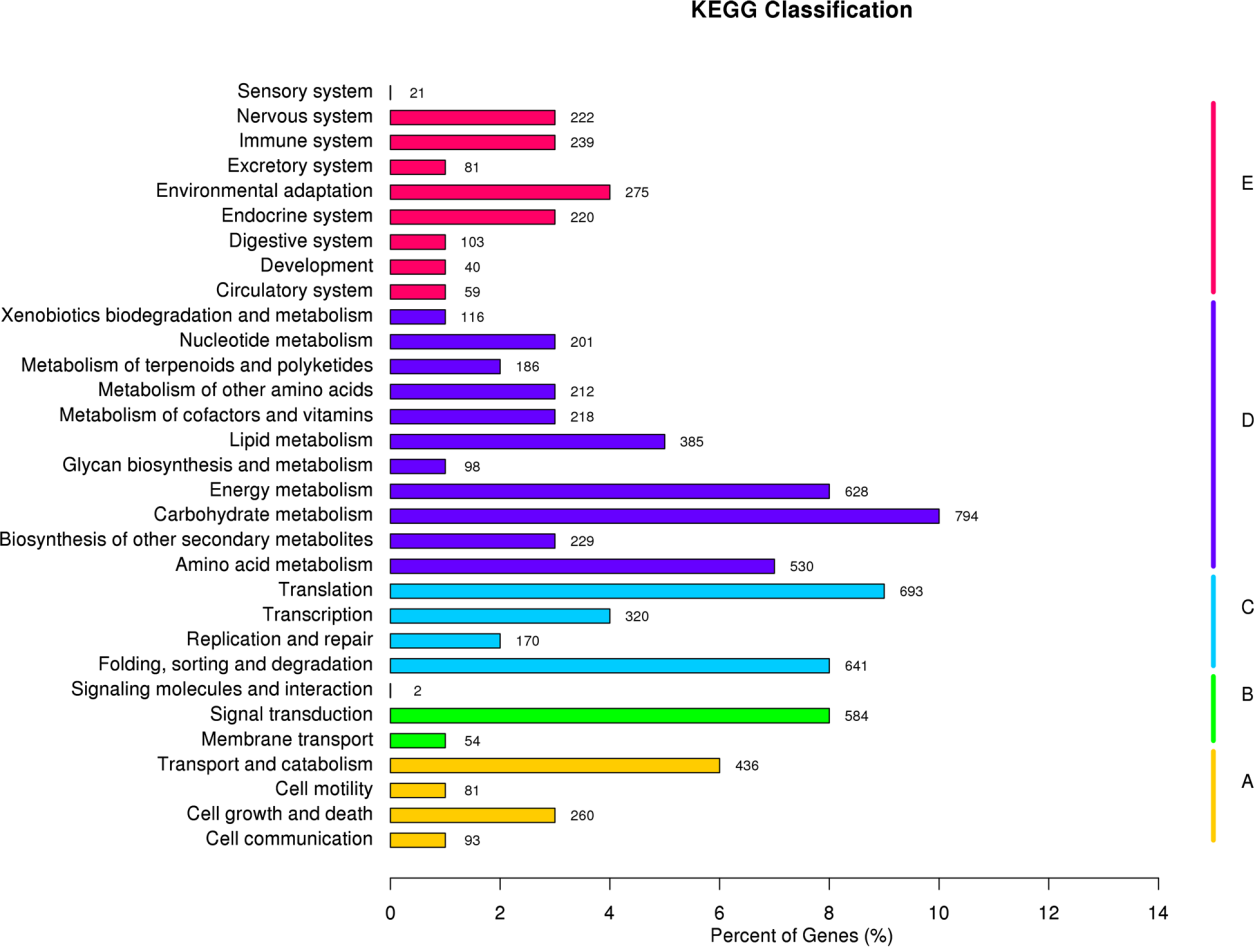
Distribution of the number of genes expressed in various metabolic pathway. **a** Cellular processes; **b** Invironmental information processing; **c** Genetic information processing; **d** Metabolism; **e** Organismal systems

KEGG pathways were also searched for biological interpretation of functions of the assembled unigenes. A total of 7742 unigenes were mapped to 31 pathways (Additional file 5: Table S4, Fig. 5). As shown in Fig. 5, the majority of unigenes were classified into pathways for carbohydrate metabolism (794), translation (693), folding, sorting and degradation (641), and energy metabolism (628). In contrast, few unigenes were found in some pathways, for example, signaling molecules and interaction (2), and sensory system (21).

### Global analysis of gene expression in IZE, IZE_Suc and SE_5w

To characterize the differences of molecular response among IZE, IZE_Suc and SE_5w, expression levels of the assembled unigenes were calculated by RPKM using RSEM software [37]. The three samples showed similar RPKM density distribution (Additional file 7: Figure S2). The results showed that only a small proportion of genes are highly expressed (Table 3). Based on the values of RPKM, approximately 31432 (82.67 % of the 38020, RPKM > 0.3) annotated unigenes showed ubiquitous expression in all the three samples. A total of 44323, 57917 and 49364 genes showed expression (RPKM > 0.3, with 95 % confidence) in IZE, IZE_Suc and SE_5w samples, respectively. 1851 (1.62 %), 1866 (1.63 %) and 1666 (1.46 %) genes (>60 RPKM) were highly expressed in IZE, IZE_Suc and SE_5w, respectively (Table 3). The top 10 most expressed genes in IZE, IZE_Suc and SE_5w had high RPKM values ranging from 2142 to 6688, 1433 to 4452, and 3757 to 23062, respectively. The top 20 most expressed genes from the three libraries are shown in Tables 4, 5 and 6.

**Table 3:** Gene expression values for the samples of IZE, IZE_Suc and SE_5w of camphor tree given in RPKM

RPKM Interval	IZE	IZE_Suc	SE_5w
0–0.1	38793(33.96 %)	24200(21.19 %)	35254(30.86 %)
0.1–0.3	31113(27.24 %)	32112(28.11 %)	29629(25.94 %)
0.3–3.57	29099(25.47 %)	42289(37.02 %)	34284(30.01 %)
3.57–15	8305(7.27 %)	8180(7.16 %)	8301(7.27 %)
15–60	5068(4.44 %)	5582(4.89 %)	5095(4.46 %)
>60	1851(1.62 %)	1866(1.63 %)	1666(1.46 %)

**Table 4:** The top 20 most expressed genes from IZE library

Gene id	RPKM	Description
comp93556_c0	6688.14	Serine/threonine protein kinase
comp93739_c0	5087.09	Metallothionein-like protein 2a [Nelumbo nucifera]
comp96566_c0	4146.24	RhoA GTPase effector DIA/Diaphanous
comp110416_c0	3628.56	Xyloglucan endotransglucosylase/hydrolase [Rosa hybrid cultivar]
comp96556_c0	2666.02	Polyubiquitin [Cicer arietinum]
comp108680_c0	2444.65	AP2/ERF domain-containing transcription factor [Populus trichocarpa]
comp100175_c0	2423.43	PREDICTED: uncharacterized protein LOC100255094 [Vitis vinifera]
comp101234_c0	2370.67	Late embryogenesis abundant protein [Sesuvium portulacastrum]
comp103367_c0	2199.46	Tubulin alpha-7 chain [Medicago truncatula]
comp57982_c0	2142.17	Glycine-rich RNA-binding protein GRP1A [Sinapis alba]
comp101258_c0	2040.98	Bowman-Birk type proteinase inhibitor [Lupinus albus]
comp110065_c1	1625.20	Cytochrome P450, putative [Ricinus communis]
comp109418_c0	1523.87	Heat-shock protein, putative [Ricinus communis]
comp30496_c0	1483.98	Translationally controlled tumor protein [Hevea brasiliensis]
comp97314_c0	1478.90	Hypothetical protein VITISV_035079 [Vitis vinifera]
comp101235_c0	1448.72	Annexin [Gossypium hirsutum]
comp109861_c0	1435.23	Heat shock cognate 70 kDa protein isoform 1 [Vitis vinifera]
comp110306_c3	1433.02	ADP-ribosylation factor [Medicago truncatula]
comp110306_c2	1345.93	Umecyanin [Armoracia rusticana]
comp109340_c0	1341.55	Cytochrome P450 89A2-like [Vitis vinifera]

**Table 5:** The top 20 most expressed genes from IZE_Suc library

Gene id	RPKM	Description
comp87631_c0	4452.17	Alcohol dehydrogenase 1 [Solanum tuberosum]
comp98297_c0	3790.17	Thioredoxin H-type [Medicago truncatula]
comp57982_c0	3444.90	Glycine-rich RNA-binding protein GRP1A [Sinapis alba]
comp93739_c0	2837.91	Metallothionein-like protein 2a [Nelumbo nucifera]
comp96556_c0	2314.52	Polyubiquitin [Cicer arietinum]
comp96883_c0	2051.84	Probable glutathione S-transferase [Vitis vinifera]
comp93556_c0	1792.74	Serine/threonine protein kinase
comp103430_c1	1727.23	Probable aquaporin PIP2-5-like [Glycine max]
comp110306_c3	1515.34	ADP-ribosylation factor [Medicago truncatula]
comp103367_c0	1432.87	Tubulin alpha-7 chain [Medicago truncatula]
comp97309_c0	1377.05	Glyceraldehyde-3-phosphate dehydrogenase, cytosolic [Magnolia liliiflora]
comp96172_c0	1376.65	Glyceraldehyde-3-phosphate dehydrogenase, partial [Eriobotrya japonica]
comp30496_c0	1325.62	Translationally controlled tumor protein [Hevea brasiliensis]
comp86932_c0	1297.76	Fructose-bisphosphate aldolase [Persea americana]
comp94640_c0	1253.98	Stem-specific protein TSJT1 [Vitis vinifera]
comp110306_c2	1231.54	Umecyanin [Armoracia rusticana]
comp87522_c0	1210.82	48 kDa dehydrin-like protein [Cornus sericea]
comp110467_c0	1204.51	Conserved hypothetical protein [Ricinus communis]
comp86219_c0	1202.35	GTP-binding nuclear protein Ran1 [Solanum lycopersicum]
comp108123_c1	1194.13	Pyruvate decarboxylase 1 [Lotus corniculatus]

**Table 6:** The top 20 most expressed genes from SE_5w library

Gene id	RPKM	Description
comp108950_c0	23061.85	7S globulin [Sesamum indicum]
comp93739_c0	11305.68	Metallothionein-like protein 2a [Nelumbo nucifera]
comp90165_c0	10427.33	--
comp30459_c0	8028.17	Oleosin H-isoform [Ficus pumila var. awkeotsang]
comp93572_c2	7081.29	Glutathione S-transferase [Chimonanthus praecox]
comp90198_c0	6050.84	Putative defensin 1 [Aquilegia pyrenaica]
comp94419_c1	5315.23	--
comp98280_c0	4237.01	--
comp57982_c0	3871.00	Glycine-rich RNA-binding protein GRP1A [Sinapis alba]
comp101235_c0	3756.90	Annexin [Gossypium hirsutum]
comp93556_c0	3306.89	Serine/threonine protein kinase
comp101258_c0	2908.99	Bowman-Birk type proteinase inhibitor [Lupinus albus]
comp96556_c0	2422.08	Polyubiquitin [Cicer arietinum]
comp79025_c0	2222.47	Pollen allergen Pla o 3 [Platanus orientalis]
comp98505_c1	2159.77	36.4 kDa proline-rich protein [Solanum lycopersicum]
comp30496_c0	2015.98	Translationally controlled tumor protein [Hevea brasiliensis]
comp30462_c0	1943.65	Translationally-controlled tumor protein homolog [Oryza sativa subsp. Japonica]
comp87719_c1	1837.68	Elongation factor 1-alpha [Manihot esculenta]
comp90471_c0	1715.29	Chitinase C [Ananas comosus]
comp110306_c3	1632.62	ADP-ribosylation factor [Medicago truncatula]

**Fig. 6:**
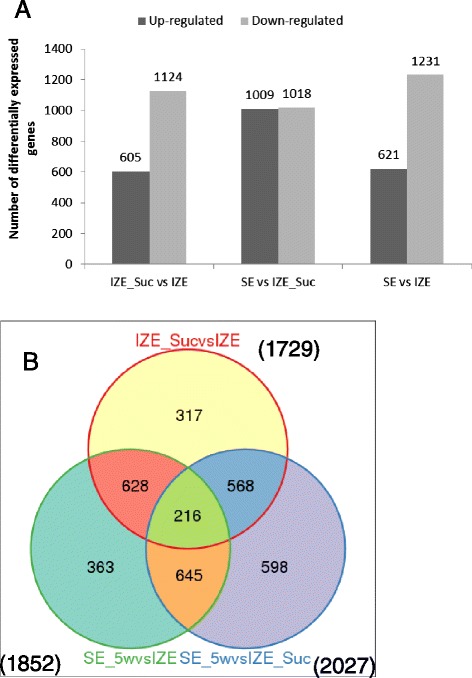
Venn diagram and Histogram of gene numbers differentially expressed during sucrose treatment and somatic embryo formation in camphor tree. **a** Histogram diagram showing the number of DEGs up- or down-regulated between different libraries; **b** A Venn diagram for analysis of the number of differentially expressed genes from IZE_Suc vs IZE, SE_5w vs IZE, and SE_5w vs IZE_Suc

To characterize the differentially expressed genes (DEGs) during somatic embryogenesis initiation in camphor tree, 3335 DEGs were singled out by comparing the three libraries in pairs (Additional file 8: Figure S3). As shown in Fig. 6a, more genes were down-regulated than up-regulated in the process of somatic embryogenesis. Between each two libraries, IZE_Suc vs IZE, SE_5w vs IZE_Suc and SE_5w vs IZE, 1729, 2027 and 1852 unigenes were differentially expressed, respectively (Additional file 9: Table S6). Of the DEGs, 216 unigenes were differentially expressed in all the three comparisons, 317 unigenes were specifically differential expressed between IZE and IZE_Suc, 598 between SE and IZE_Suc, and 363 between SE and IZE (Fig. 6b). These results indicated that osmotic stress pretreatment of IZE and culture of IZE_Suc on somatic embryo induction medium caused significant differential gene expression.

**Fig. 7:**
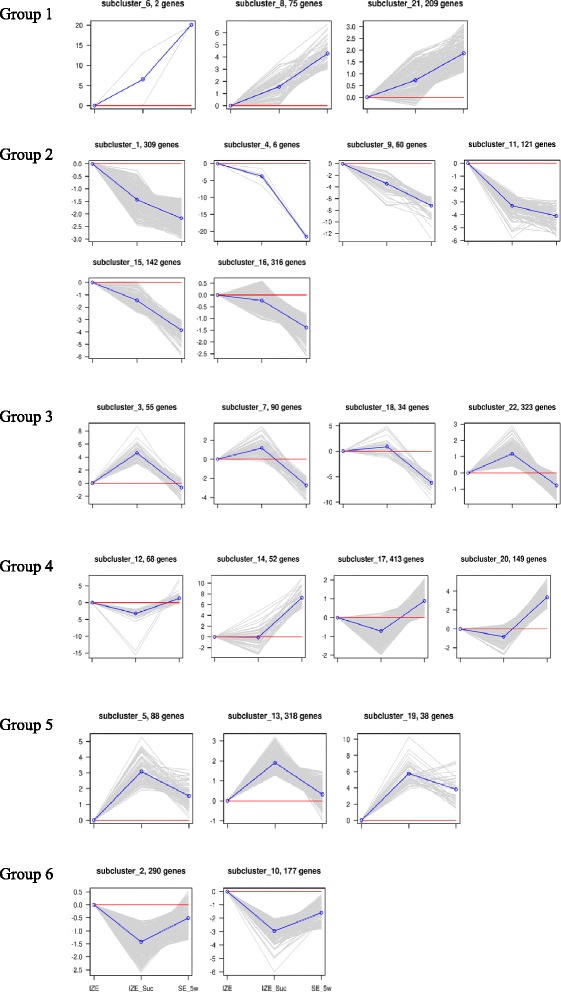
Cluster analysis of differentially expressed genes during somatic embryo induction in camphor tree based on K-means method

To further investigate the expression profiles of the DEGs, they were divided into 6 groups based on the results of K-means clustering (Fig. 7). Group 1 and 2 contained genes positively or negatively modulated during the whole process of somatic embryo initiation and formation. Group 3 contained genes positively modulated during sucrose pretreatment, and then negatively modulated in the process of somatic embryo formation, while group 4 contained genes modulated in the opposite way in both processes. Genes in group 5 were up-regulated after sucrose pretreatment, and then down-regulated during somatic embryo formation, whose expressions were still higher than that of the initial IZE explant. Genes expressed with the opposite pattern of group 5 fell into group 6.

### Functional annotation of differentially expressed genes

**Fig. 8:**
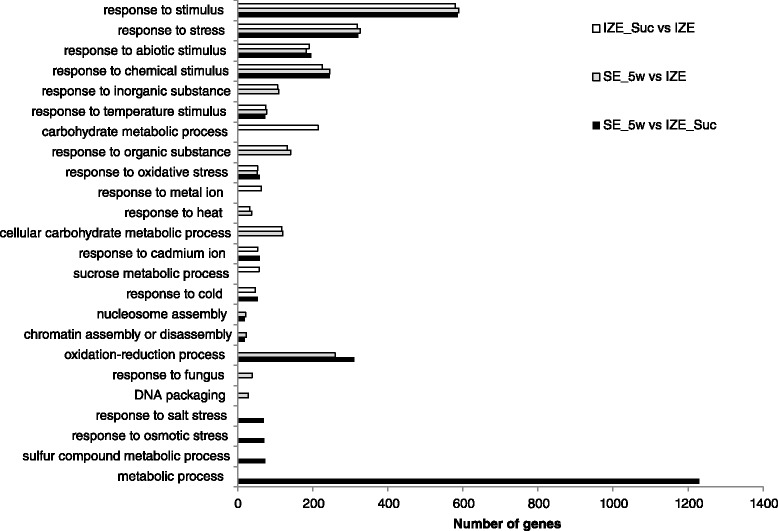
Functional categorization of genes differentially expressed during sucrose pretreatment and somatic embryo iniation based on biological process of Gene Ontology

GO enrichment analysis for the DEGs was conducted to characterize the expression changes in the three samples with the whole transcriptome dataset as the background. The DEGs were assigned to 24 GO categories based on biological process (Fig. 8). The result showed that “response to stimulus”, “response to stress”, “response to abiotic stimulus” and “response to chemical stimulus” were among the most highly represented groups in the biological process category in the process of somatic embryogenesis. Other biological processes such as “oxidation-reduction process”, “metabolic process”, and “response to inorganic substance” were also identified. Some GO terms were identified only in specific comparison pair, for example, “carbohydrate metabolic process” and “sucrose metabolic process” were identified only in DEGs of IZE_Suc and IZE, while “response to salt stress”, “response to osmotic stress”, “sulfur compound metabolic process” and “metabolic process” were identified only in DEGs of SE_5w vs IZE_Suc. Among all the assigned DEGs, 1230 genes (SE_5w vs IZE) involved in “metabolic process” fell into the most highly represented group. The top ten GO terms (based on biological process) of up-regulated or down-regulated DEGs in the three comparisons are shown in Additional file 10: Table S7.

All the DEGs were mapped to terms in KEGG database to search for metabolic or signal transduction pathways in which genes were significantly enriched, compared with the whole transcriptome background. In total, 3437 DEGs were assigned to 193 KEGG pathways, of which, 1061 DEGs (IZE_Suc vs IZE) were assigned to 172 pathways, 1345 DEGs (SE_5w vs IZE_Suc) to 181 pathways, and 1031 DEGs (SE_5w vs IZE) to 157 pathways, respectively. Pathway enrichment analysis revealed that the annotated changes between IZE_Suc and IZE were mainly involved in glycolysis/gluconeogenesis, starch and sucrose metabolism, plant hormone signal transduction, PPAR signaling pathway, plant-pathogen interaction, while the annotated changes between SE_5w vs IZE were mainly involved in plant hormone signal transduction, flavonoid biosynthesis, antigen processing and presentation, plant-pathogen interaction (Additional file 11: Table S8, Additional file 12: Figure S4).

### Confirmation of somatic embryogenesis-related DEGs by qRT-PCR

**Fig. 9:**
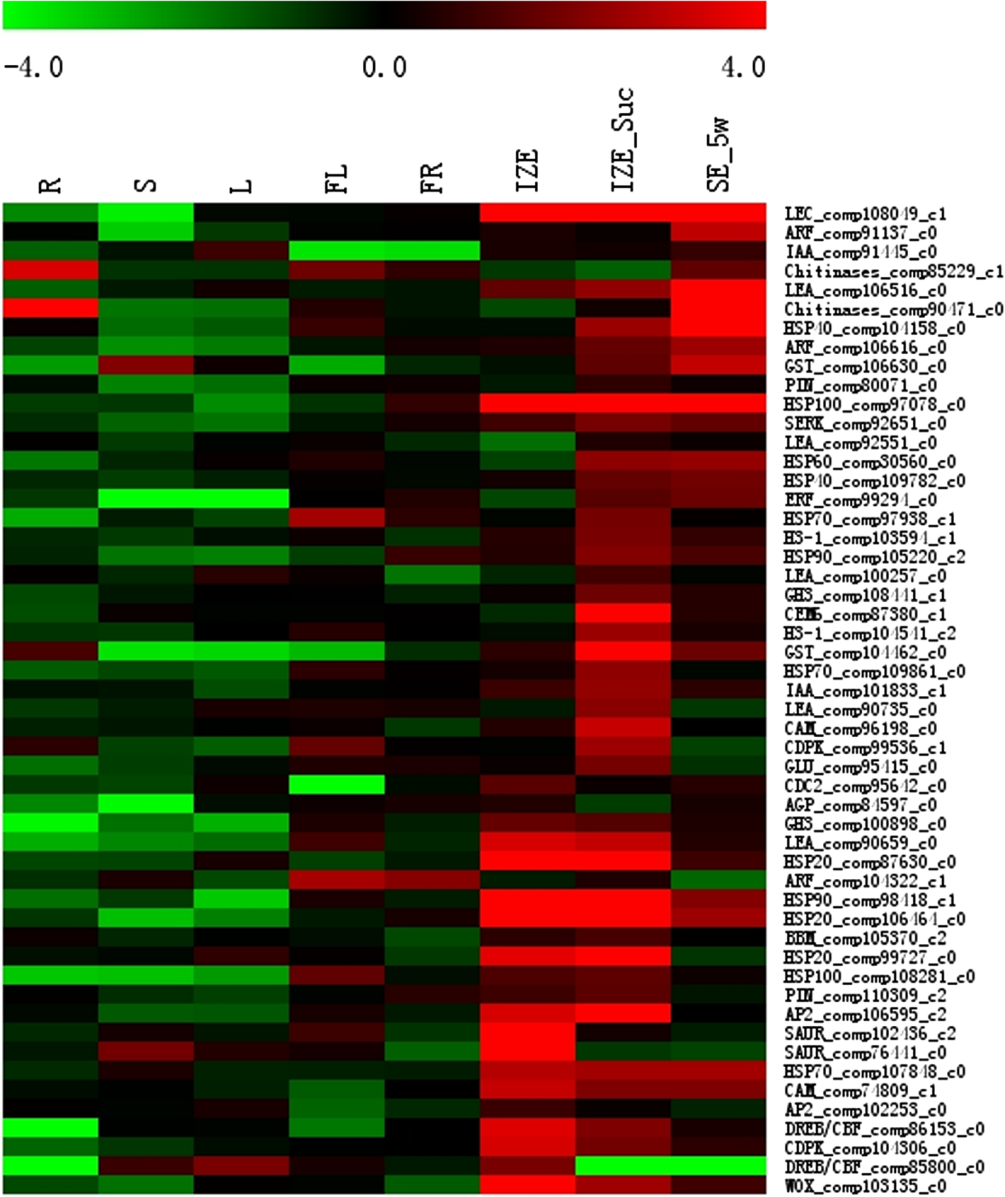
Expression patterns of 52 somatic embryogenesis-related DEGs in different tissues. The bar represents the scale of relative expression levels of DEGs, and the colors indicate relative signal intensities of DEGs. R roots, S stems, L young leaves, FL young flowers, FR young fruits, IZE immature zygotic embryos, IZE_Suc IZE pretreated with 1.0 M sucrose solution, SE_5w somatic embryos obtained from induction medium after culture for 5 weeks

With regard to the 897 somatic embryogenesis-related genes, expression data were shown in Additional file 13: Table S9 and summarized in Additional file 6: Table S5. To validate gene expression profiles obtained by RNA-seq, 52 DEGs related to somatic embryogenesis were selected for qRT-PCR analysis across eight different tissues of camphor tree: roots, stems, young leaves, young flowers, young fruits, IZE, IZE_Suc and SE_5w. The corresponding primers are listed in Additional file 2: Table S1. Based on the analyzed qRT-PCR data, all the 52 selected unigenes were expressed at varying levels in different tissues (Fig. 9). Seven unigenes, including CDC2_comp95642_c0, ERF_comp108680_c0, SAUR_comp102436_c2, SAUR_comp76441_c0, DREB/CBF_comp85800_c0, SMP_comp103536_c0, AIL_comp98550_c0, were highly expressed in IZE. HSP70_comp97938_c1, H3-1_comp103594_c1, HSP90_comp105220_c2, GH3_comp108441_c1, CEM6_comp87380_c1, H3-1_comp104541_c2, GST_comp104462_c0, HSP70_comp109861_c0, IAA_comp101833_c1, LEA_comp90735_c0, CAM_com96198_c0, CDPK_com99536_0, and GLU_comp95415_c0 showed strong expression in IZE_Suc, but relatively low expression in IZE and SE_5w. Expression of ARF_comp91137_c0, Chitinases_comp85229_c1, Chitinases_comp90471_c0, GST_comp106630_c0, HSP40_comp104158_c0 and GLU_comp107417_c3 were high in SE_5w, while low in IZE and IZE_Suc. These results confirmed that sucrose pretreatment induced differential expression of somatic embryogenesis-related genes, and these DEGs potentially play important roles during somatic embryo induction in *C. camphora*.

**Fig. 10:**
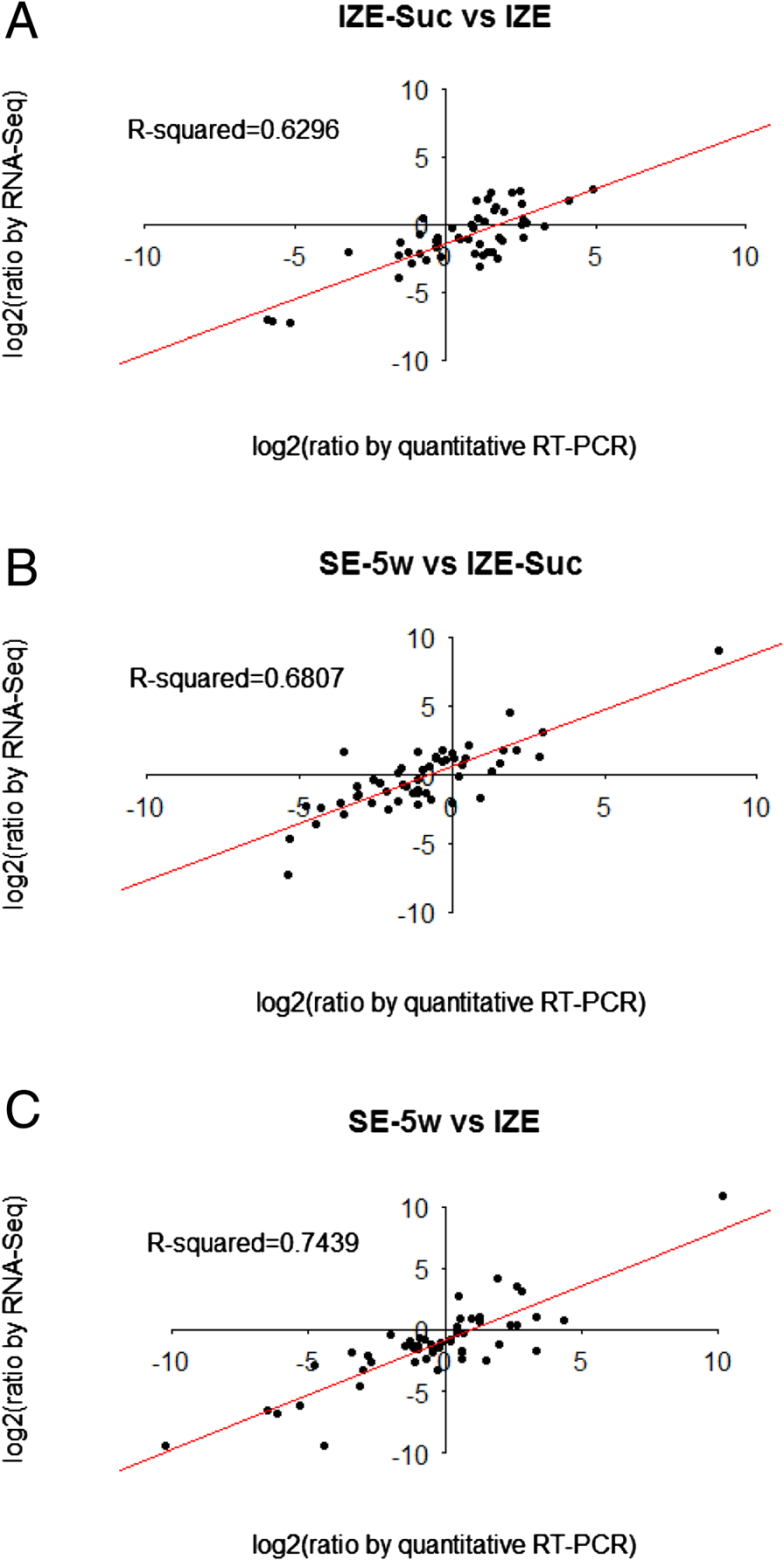
Correlation between RNA-seq and quantitative real-time PCR (qRT-PCR). Comparison of log2 fold change of 52 DEGs related to somatic embryogenesis obtained by RNA-seq and qRT-PCR for IZE_Suc vs IZE (**a**) SE_5w vs IZE_Suc (**b**) and SE_5w vs IZE (**c**) respectively

The pearson correlation coefficient was calculated by SPSS to assess the correlation between the platforms of RNA sequencing and qRT-PCR. When the comparisons of IZE_Suc versus IZE, SE_5w versus IZE_Suc, and SE_5w versus IZE were performed, gene expression levels estimated by qRT-PCR were moderately or strongly correlated with RNA-Seq results (R^2^ = 0.6296, 0.6807 and 0.7439, respectively, correlation is significant at the 0.01 level), indicating the reliability of the RNA-seq analysis (Fig. 10).

## Discussion

Camphor tree is a non-model organism lacking genome and transcriptome resources. Though somatic embryogenesis-related genes have been extensively characterized in Arabidopsis, none have been published in camphor tree. In addition, the reported transcriptome analyses of somatic embryogenesis were mainly about plant hormone induced systems, few were about stress induced, particularly sucrose stress induced somatic embryogenesis. This study provides a *de novo* assembled transcriptome and comprehensive gene expression data during somatic embryogenesis induced by sucrose pretreatment. Gene expression changes during SE initiation and formation in camphor tree were focused in this study.

### *De novo* transcriptome analysis of early somatic embryogenesis in camphor tree

Recalcitrance of plant explants to SE induction has long been an impediment to in vitro morphogenesis, which is especially relevant to woody species [43]. In camphor tree, various inducers including different plant hormones and carbon sources, used alone or in combination, failed to initiate efficient SE formation from IZEs, young leaves or flowers. Instead, we found SEs can be efficiently induced from IZEs by sucrose pretreatment without involvement of plant hormones [3]. The previous study indicated sucrose pretreatment other than plant hormones was the key factor for SE initiation in camphor tree. Such kind of direct somatic embryogenic system without exogenous application of plant hormones is invaluable in elucidating early regulatory events in embryo development [44]. As such, the explants of IZE, IZE_Suc, and the obtained SE_5w were selected for this study. Comparative transcriptome analysis of these samples provided an opportunity to examine the molecular aspects underlying early SE development.

Next-generation sequencing technology is especially suitable for gene expression profiling in somatic embryogenesis in such species. As a platform allowing generation of massive amounts of genomic resources rapidly and cost-effectively, this technology has already been used in transcriptome analysis of somatic embryogenesis in cotton [26, 45], hybrid yellow poplar [46], Japanese larch [47], *Lycoris aurea* [48], Longan [49, 50], and maize [51]. Before this study, only a few number of nucleotide sequences and ESTs of camphor tree were deposited in the NCBI database. Here we report a comprehensive analysis of transcriptome dynamics that may serve as a gene expression profile blueprint in somatic embryogenesis of camphor tree. One of our main goals was to adapt the RNA-Seq technology to this notable development process and to analyze the gene expression profile. *De novo* transcriptome assembly of the IZE, IZE_Suc and SE_5w by Illumina HiSeq 2000 resulted in a large amount of sequence and gene expression information. Molecular analysis was conducted to gain understanding of key events underlying the process of SE initiation in camphor tree. These sequences provided abundant information for further studies of somatic embryogenesis in camphor tree.

### Differentially expressed somatic embryogenesis-related genes during embryogenic initiation

Somatic embryogenesis is a process during which genes were selectively expressed. In the present study, numerous somatic embryogenesis-related genes, including genes responsible for cell cycle and cell wall, hormone-responsive genes, genes in signal transduction pathway in somatic embryogenesis, and transcription factors were differentially expressed during SE induction (Fig. 9). Up-regulated expression of genes from families of *GH3*, *PIN*, *Indoleacetic Acid-Induced Protein* (*Aux/IAA*), *ARF*, *HSP*, *Late Embryogenesis Abundant* (*LEA*), *CEM6*, *H3-1*, *SERK*, *Calmodulin* (*CAM*), *Calcium-Dependent Protein Kinase* (*CDPK*), *BBM*, *Apetala2* (*AP2*), and *ERF* was observed after sucrose treatment.

Exposing excised plant tissues to in vitro culture conditions containing high concentrations of auxin is the most used strategy to elicit somatic embryogenesis. Changes of gene expression have been observed in auxin-induced somatic embryogenesis via investigating the role of auxin signaling [52]. Auxin surges occurred in the process of somatic embryogenesis, which resulted in the isolation of several corresponding gene classes, including *ARFs*, *Aux/IAAs*, *GH3s*, *PINs*, and *Small Auxin-up RNAs* (*SAURs*) [30]. In addition, *HSPs* were also found to be auxin-responsive genes during SE development. Members of the *HSP* family have been reported to be highly expressed during the initiation of somatic embryogenesis by auxin [53]. Studies have confirmed that some genes of *HSPs* were expressed during the process of somatic embryo development in carrot [54] and alfalfa [55]. In this study, sucrose pretreatment induced significantly differential expression of transcripts from these gene families. These results indicated that although no auxin was applied for SE induction in camphor tree, the auxin-related genes may still function in SE induction.

*LEA* genes are abundantly expressed in late zygotic embryogenesis in many plant species [56]. Although *LEA* genes are well known ABA-inducible, the expression of ABA-inducible genes is not necessarily correlated with the level of ABA [57]. In camphor tree, up-regulated expression of *LEA* genes were observed in IZE_Suc, indicating osmotic stress is involved in regulating the expression of *LEA* genes. It is consistent with the result that the synthesis of *LEA* proteins occurs as soon as embryogenesis is initiated, which requires the application of a stress or exogenous ABA [5].

In addition to differentially expressed genes, various signal transduction pathways for activating or repressing numerous gene sets are also involved in the process of embryogeny acquisition [7]. *SERKs*, the first one of which was isolated from carrot suspension cultures up to the globular-shaped stage of embryogenesis [58], have been detected and identified in the process of somatic embryogenesis in various species [59–63]. In the present study, a *SERK* gene was up-regulated in IZE_Suc, indicating that the *SERK* gene plays a role in mediating SE initiation in camphor tree. *CaM* and *CDPK* are two of the three major classes of Ca^2+^ sensors, which might play an intermediary role during somatic embryogenesis [64, 65]. Genes of *CaM* and *CDPK* showed differential expression patterns between IZE and IZE_Suc, suggesting their potential functions in the early stage of somatic embryogenesis in camphor tree.

Several transcription factors, including genes from *LEC*, *BBM*, *AP2*, *ERF*, *DREB*, *Wuschel-Related Homeobox* (*WOX*) families that play regulatory roles in embryogenic processes [66, 67], were also found up or down regulated in this study, which indicated they might also be critical during somatic embryogenesis.

### Differential expression of stress responsive genes during embryogenic initiation

Gene enrichment analysis showed significantly differential expression of genes responding to stress and stimulus (Fig. 8). Treating of explants with 0.5 M sucrose solution exposed the embryos to significantly different conditions from their original environment, which may induce differential expression of stress responsive genes. Plants deploy diverse molecular and cellular mechanisms to survive in stressful environments [68]. Some stress-related genes associated with early stages of SE including *GST*s, *Germin-like Proteins* (*GLPs*), *HSP*s, *Chitinases* and *β-1,3-Glucanases* [8] were also observed differentially expressed during SE induction in our RNA-seq profiling. Members of *GSTs* superfamily, playing important roles in the overall natural defense mechanisms in all living organisms, have been shown up-regulated expression during auxin-induced somatic embryogenesis in soybean [25] and cotton [69]. Five *GST* transcripts present in our DEGs (Additional file 13: Table S9) were also up-regulated in the process of sucrose treatment. *β-1,3-Glucanases* belong to a large gene family. Members of *β-1,3-Glucanases* could be induced by pathogen attack or treatment with biotic or abiotic elicitors in plants [70]. Studies on expression of *β-1,3-Glucanases* during somatic embryogenesis process of *Cichorium*, spruce, and *Araucaria angustifolia* suggested that *β-1,3-Glucanases* may have implications in the somatic embryogenesis process. We found that one *β-1,3-Glucanase* gene (comp107417_c3) was highly expressed after sucrose treatment, which was also up-regulated during the process of somatic embryo formation (Additional file 13: Table S9).

### Comparison of gene expression between somatic and zygotic embryos

SEs undergo a similar developmental program to zygotic embryos [5]. The same set of genes might be operating in both processes to specify embryo development [71]. However, some key differences exist between the two types of embryos, including the lack of surrounding embryo sac and differentiation of endosperm in somatic embryos. It has been observed somatic embryos exhibited more metabolic activity than zygotic embryos at parallel developmental stages in cotton [71]. In this study, the comparison of gene expression in SEs and zygotic embryos showed the number of down-regulated DEGs was much more than that of up-regulated DEGs in SEs. The GO annotations of DEGs revealed that genes response to stimulus or stress were significantly enriched. Secondary SE formation on the culture medium [3] and the results in the present study lead to the speculation that in vitro tissue culture conditions activate or suppress expression of stress responsive genes, which is in consistent with the suggestion that cells of cultured SEs underwent stress stimulation by exogenous compound in vitro, and SEs formation was the outcome of an in vitro adaption process to the culture environment [71].

## Conclusions

In this work, *de novo* assembled transcriptomes of three embyogenic tissues of camphor tree (IZE, IZE_Suc and SE_5w samples) were analyzed and a large amount of sequence information was obtained. Gene expression profiles in the process of SE initiation induced by sucrose treatment and SE formation were investigated. Differential expression of genes potentially functioned in acquisition of embryogenic competence and stress response are of particularly interest. Differential expression of somatic embryogenesis-related genes (IZE_Suc vs IZE) indicates that sucrose induced somatic embryogenesis may share or partly share the mechanisms of somatic embryogenesis induced by plant hormones. This study provides new information about gene expression at early somatic embryogenesis stage, and meanwhile provides comprehensive gene expression data for camphor tree somatic embryogenesis that could serve as an important platform resource for further functional studies in plant embryogenesis.

### Availability of supporting data

The Illumina sequence data from this study have been submitted as BioProject ID [PRJNA288748] to the NCBI sequence read archive under the accession number [SRP060394]. All the supporting data are included as additional files.

## Additional files

Additional file 1: Figure S1. Samples collected for transcriptome sequencing. (DOCX 470 kb) Additional file 2: Table S1. Gene specific primers used in qRT-PCR. (XLSX 18 kb) Additional file 3: Table S2. Length and number distribution of the unigenes. (XLSX 10 kb) Additional file 4: Table S3. Functional annotation of non-redundant unigenes against Nr, Nt, Swiss-Prot, PFAM, GO and KOG databases. (XLSX 19135 kb) Additional file 5: Table S4. Functional annotation of non-redundant unigenes against KEGG database. (XLSX 66 kb) Additional file 6: Table S5. Expression of somatic embryogenesis-related genes in IZE, IZE_Suc and SE_5w in camphor tree based on the results of RNA-Seq. (XLSX 11 kb) Additional file 7: Figure S2. Frequency distribution of IZE, IZE_Suc and SE_5w by RPKM. (DOCX 222 kb) Additional file 8: Figure S3. Volcano plot of differential gene expression in IZE_Suc vs IZE, ZE_5w vs IZE_Suc and SE_5w vs IZE. (DOCX 256 kb) Additional file 9: Table S6. List of DEGs singled out by comparing the libraries of IZE_Suc vs IZE, SE_5w vs IZE_Suc and SE_5w vs IZE. (XLSX 382 kb) Additional file 10: Table S7. The top 10 GO terms of up-regulated and down-regulated DEGs in the three comparisons based on biological process. (XLSX 13 kb) Additional file 11: Table S8. KEGG enrichment analysis of DEGs. (XLSX 19 kb) Additional file 12: Figure S4. Scatterplot of DEG enriched KEGG pathway in IZE_Suc vs IZE, ZE_5w vs IZE_Suc and SE_5w vs IZE. (DOCX 145 kb) Additional file 13: Table S9. Differentially expressed DEGs associated with somatic embryogenesis. (XLSX 58 kb)

## Abbreviations

BLAST: Basic local alignment search tool
CTAB: Cetyltrimethyl ammonium bromide
DEG: Differentially expressed gene
GO: Gene ontology
IZE: Immature zygotic embryo
KEGG: Kyoto encyclopedia of genes and genomes
KOG/COG: Clusters of orthologous groups
NCBI: National center for biotechnology information
Nr: Non-redundant database
Nt: Nucleotide database
qRT-PCR: Quantitative Real-Time PCR
RIN: RNA integrity number
RPKM: Reads per kilo bases per million mapped reads
SE: Somatic embryo
SPSS: Statistical product and service solutions
