# Supplementary material for: De novo comparative transcriptome analysis provides new insights into sucrose induced somatic embryogenesis in camphor tree (Cinnamomum camphora L.)
Source: BMC Genomics. 2016 Jan 5;17:26. doi: 10.1186/s12864-015-2357-8 (PMC4700650; doi:10.1186/s12864-015-2357-8)
Supplement: Additional file 1: Figure S1. — Samples collected for transcriptome sequencing. (DOCX 470 kb) [file 12864_2015_2357_MOESM1_ESM.docx]

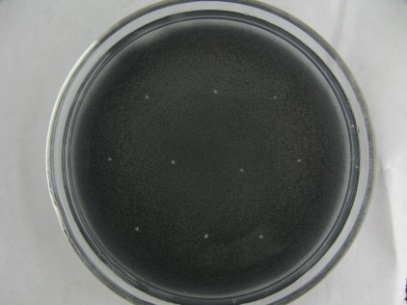

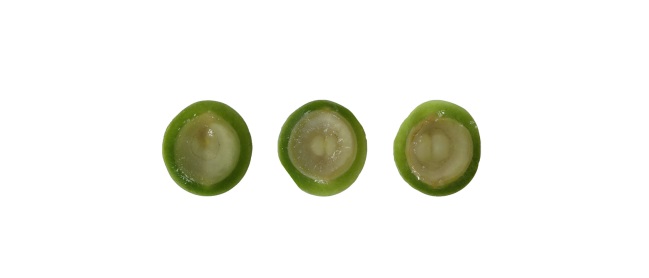

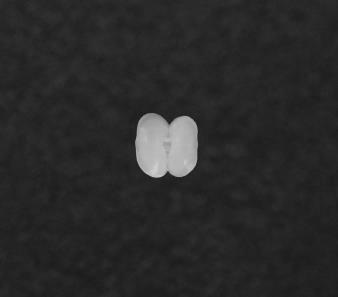

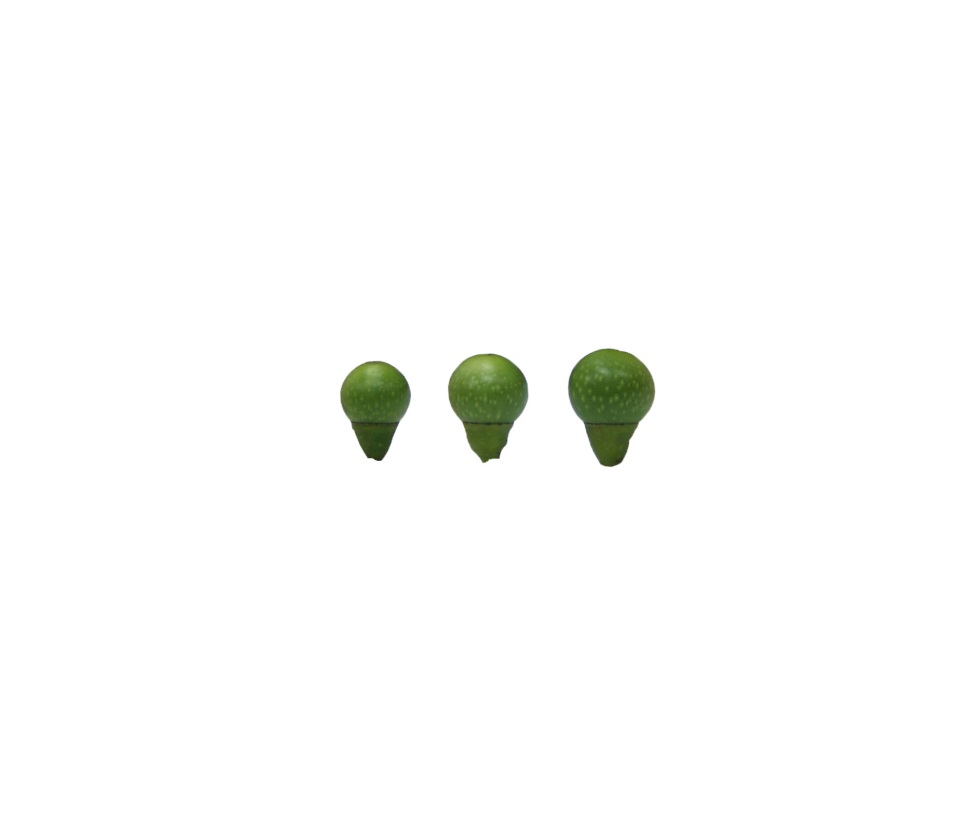


**A**

**B**

J

**C**


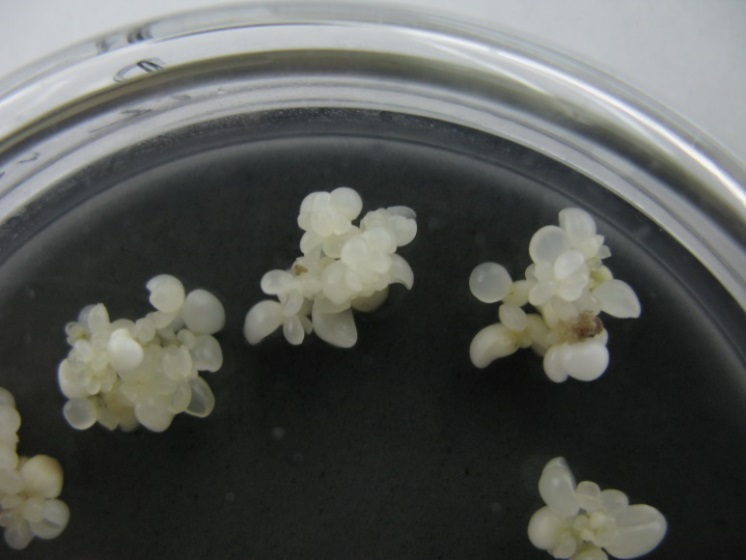


**E**

**D**

**Additional file 1: Figure S1 Samples collected for transcriptome sequencing.** **A** The immature fruit of camphor tree collected 11-12, 12-13 and 13-14 weeks after open pollination (from left to right), respectively. Red dotted line indicated the cut position for IZE isolation; **B** The immature fruits respectively collected 11-12, 12-13 and 13-14 weeks after open pollination (from left to right) were cut, and IZEs (black arrow) were visible at the distal end; **C** Close view of one IZE from the immature fruit collected 12-13 weeks after open pollination; **D** IZE_Suc were cultured on SE induction medium after treated with 0.5 M sucrose solution for 72 h; **E** SE_5w obtained from IZE_Suc after cultured for five weeks on induction medium. **A B C** bars = 1 mm; **D E** bars = 1cm.
