# Supplementary material for: De novo comparative transcriptome analysis provides new insights into sucrose induced somatic embryogenesis in camphor tree (Cinnamomum camphora L.)
Source: BMC Genomics. 2016 Jan 5;17:26. doi: 10.1186/s12864-015-2357-8 (PMC4700650; doi:10.1186/s12864-015-2357-8)
Supplement: Additional file 7: Figure S2. — Frequency distribution of IZE, IZE_Suc and SE_5w by RPKM. (DOCX 222 kb) [file 12864_2015_2357_MOESM7_ESM.docx]

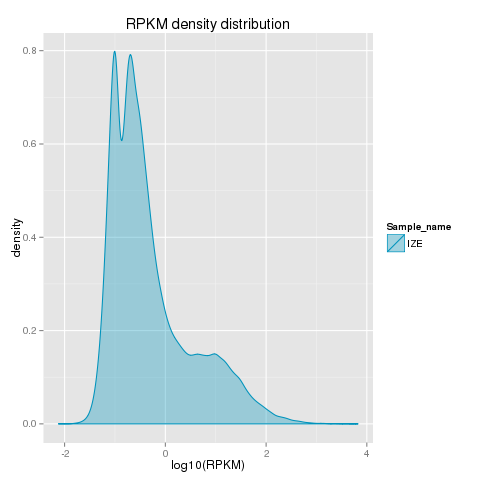


A


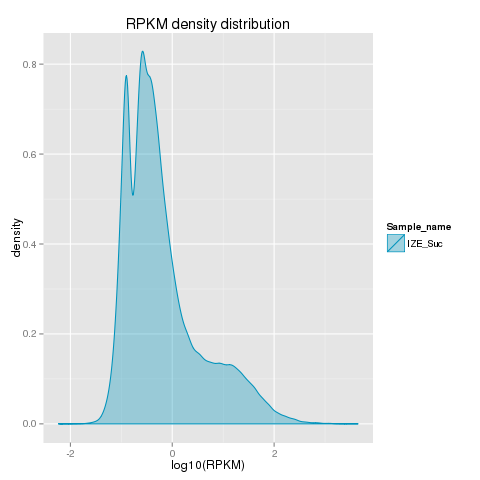


B


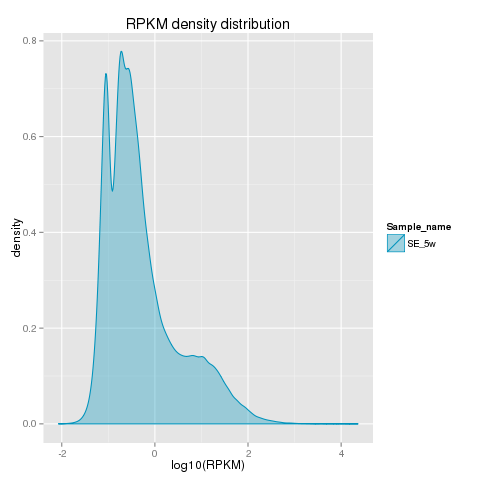


C

**Additional File 7: Figure S2. Frequency distribution of IZE (A), IZE_Suc (B) and SE_5w (C) by RPKM.** The x-axis indicates the value of log10(RPKM), and the y-axis indicates the density of log10(RPKM) value.
