# Supplementary material for: De novo comparative transcriptome analysis provides new insights into sucrose induced somatic embryogenesis in camphor tree (Cinnamomum camphora L.)
Source: BMC Genomics. 2016 Jan 5;17:26. doi: 10.1186/s12864-015-2357-8 (PMC4700650; doi:10.1186/s12864-015-2357-8)
Supplement: Additional file 8: Figure S3. — Volcano plot of differential gene expression in IZE_Suc vs IZE, ZE_5w vs IZE_Suc and SE_5w vs IZE. (DOCX 256 kb) [file 12864_2015_2357_MOESM8_ESM.docx]

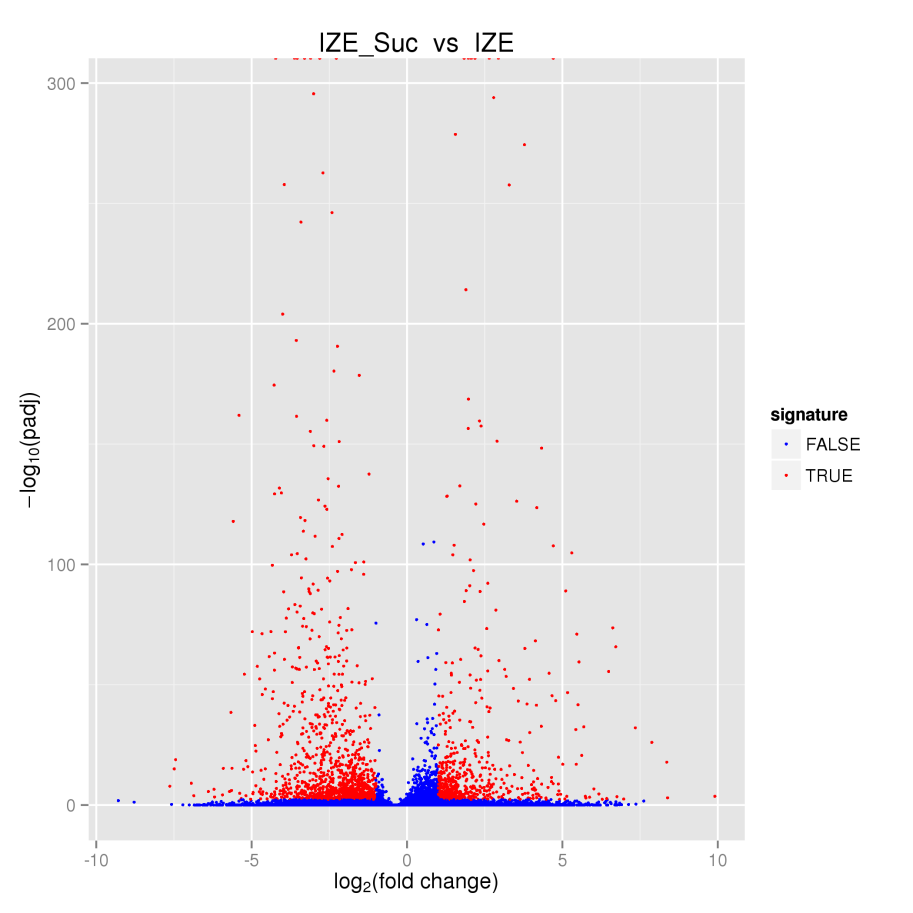

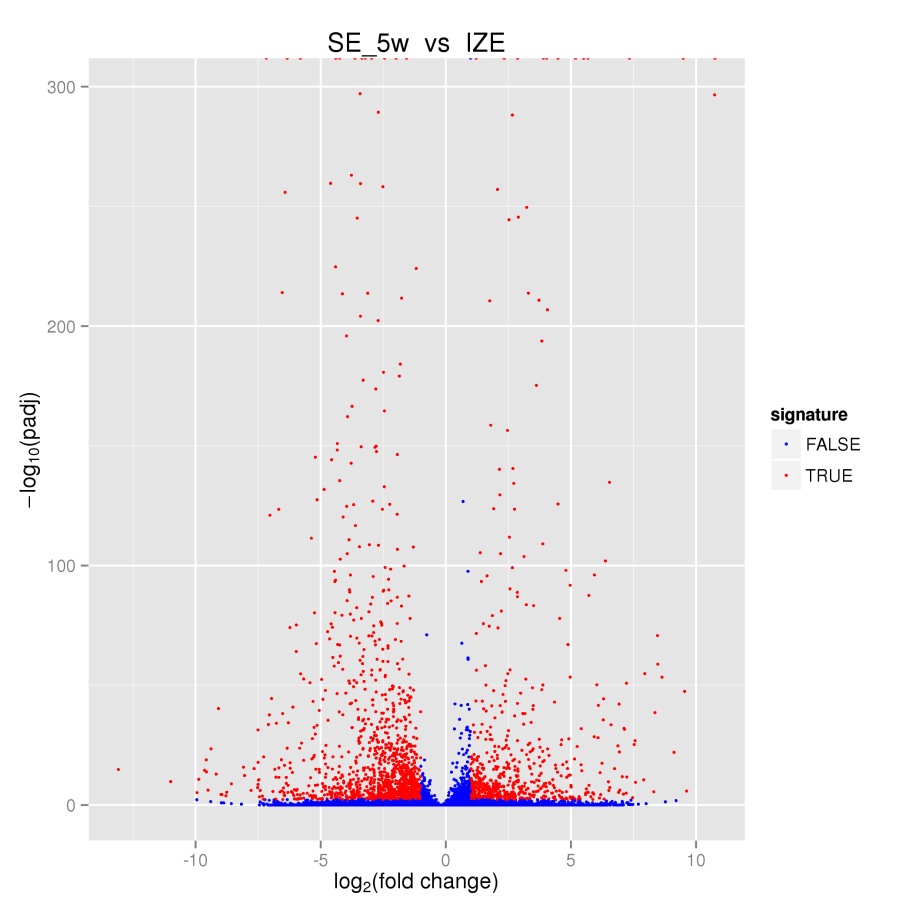

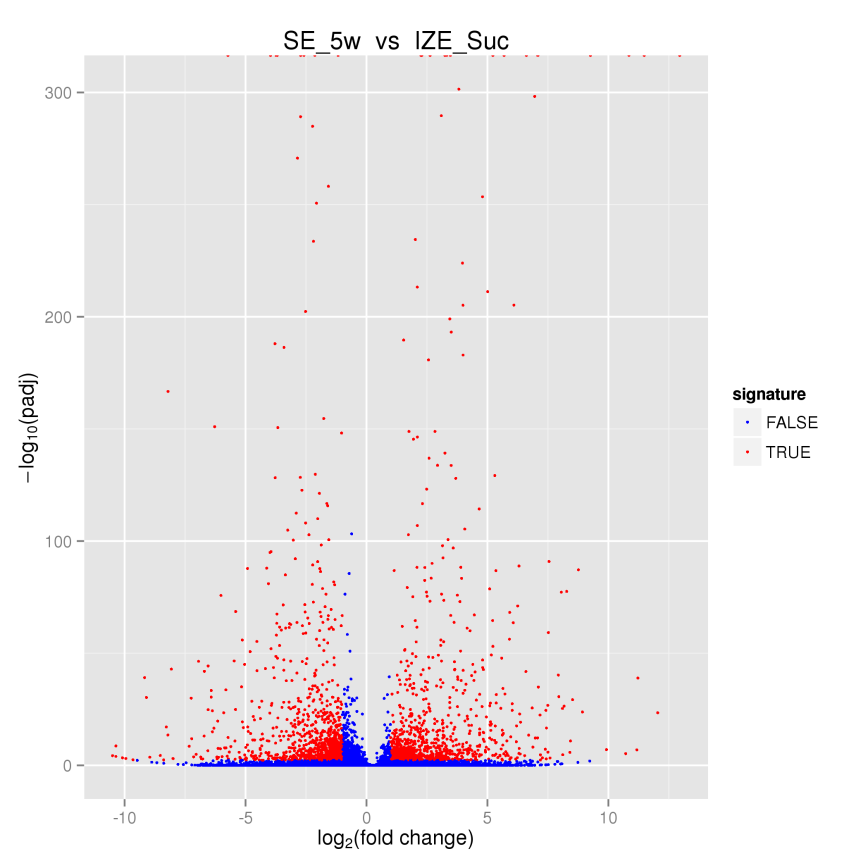


B

A

**Additional file 8: Figure S3. Volcano plot of differential gene expression in IZE_Suc vs IZE (A), ZE_5w vs IZE_Suc (B) and SE_5w vs IZE (C).** The fold change (group 1/group 2) of DEGs was relative to their expression in the three comparisons. DEGs with a positive fold change were up-regulated in group 1 (down-regulated in group 2), and DEGs with a negative fold change were down-regulated in group 1 (up-regulated in group 2). DEGs that were statistically significant (q-value<0.005, log_2_fold change >1) are shown in red, while that not statistically significant are shown in blue.

C
