# Supplementary material for: De novo comparative transcriptome analysis provides new insights into sucrose induced somatic embryogenesis in camphor tree (Cinnamomum camphora L.)
Source: BMC Genomics. 2016 Jan 5;17:26. doi: 10.1186/s12864-015-2357-8 (PMC4700650; doi:10.1186/s12864-015-2357-8)
Supplement: Additional file 12: Figure S4. — Scatterplot of DEG enriched KEGG pathway in IZE_Suc vs IZE, ZE_5w vs IZE_Suc and SE_5w vs IZE. (DOCX 145 kb) [file 12864_2015_2357_MOESM12_ESM.docx]

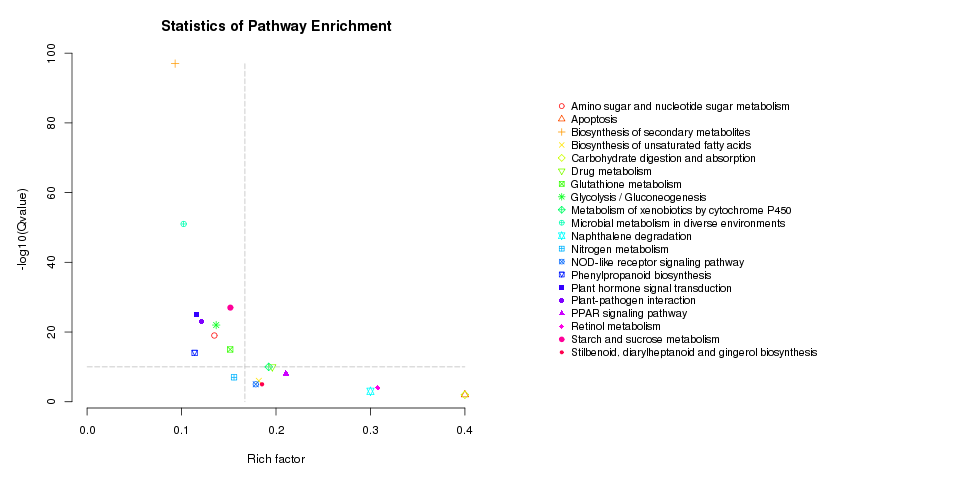


A


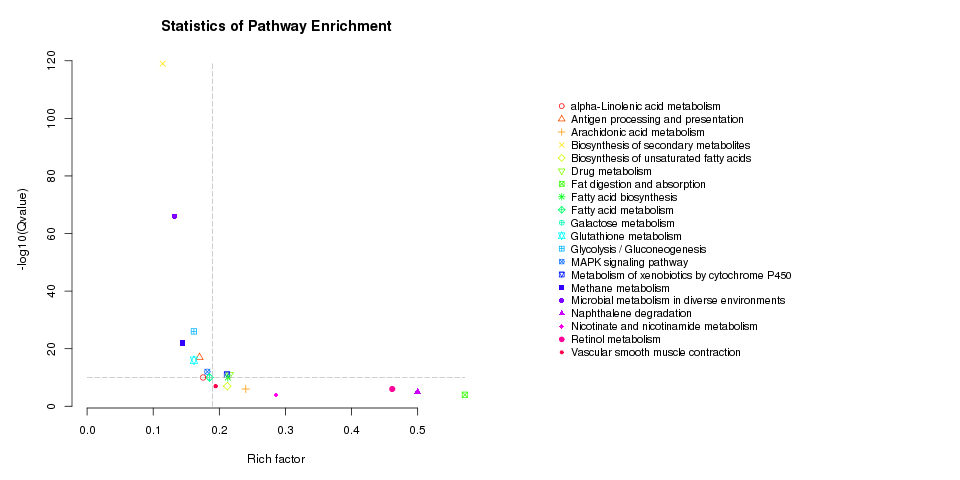


B


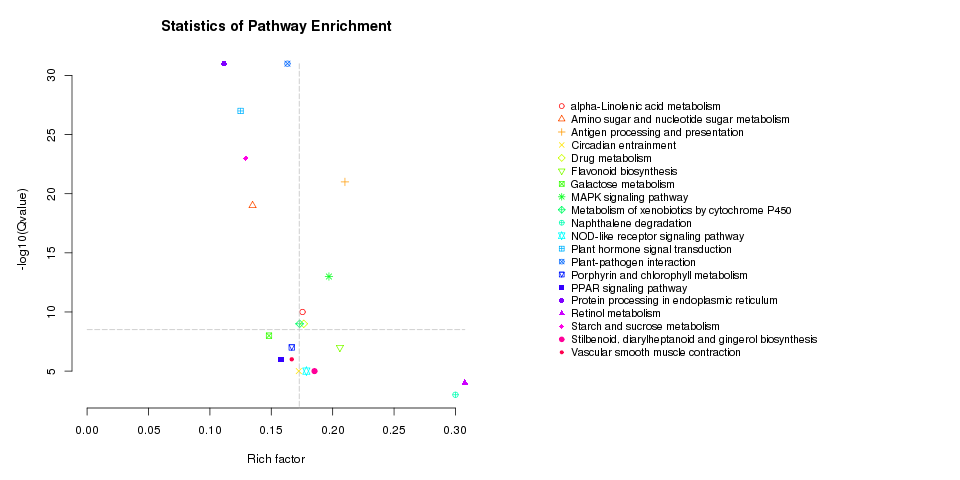


C

**Additional file 12: Figure S4. Scatterplot of DEG enriched KEGG pathway in IZE_Suc vs IZE (A), ZE_5w vs IZE_Suc (B) and SE_5w vs IZE (C).** Rich factor represents the ratio of the number of DEGs and the number of all unigenes in the pathway. Q value is the corrected p value.
